# Role of coenzyme Q_10_ as an antioxidant and bioenergizer in periodontal diseases

**DOI:** 10.4103/0253-7613.71884

**Published:** 2010-12

**Authors:** Shobha Prakash, J. Sunitha, Mayank Hans

**Affiliations:** Department of Periodontics, College of Dental Sciences, Davangere, Karnataka, India

**Keywords:** Antioxidant, bioenergizer, coenzyme Q_10_, periodontal disease

## Abstract

Periodontal disease is an inflammatory disease process resulting from the interaction of a bacterial attack and host inflammatory response. Arrays of molecules are considered to mediate the inflammatory response at one time or another, among these are free radicals and reactive oxygen species (ROS). Periodontal pathogens can induce ROS overproduction and thus may cause collagen and periodontal cell breakdown. When ROS are scavenged by antioxidants, there can be a reduction of collagen degradation. Ubiquinol (reduced form coenzyme Q_10_) serves as an endogenous antioxidant which increases the concentration of CoQ_10_ in the diseased gingiva and effectively suppresses advanced periodontal inflammation.

## 

Coenzyme Q_10_ (also known as ubiquinone) was discovered by Crane and his colleagues in 1957 in beef heart mitochondria.[1] It was first isolated from the mitochondria of bovine hearts in 1957 at the University of Wisconsin. Identification of the chemical structure and synthesis was completed by 1958. Because of its ubiquitous presence in nature and its quinone structure (similar to that of vitamin K), coenzyme Q_10_ is also known as ubiquinone.[2]

Coenzyme Q_10_ is a naturally occurring coenzyme formed from the conjugation of a benzoquinone ring with a hydrophobic isoprenoid chain of varying chain length, depending on the species.[3] The chemical nomenclature of CoQ_10_ is 2,3-dimethoxy-5-methyl-6-decaprenyl-1,4-benzoquinone that is in the trans configuration (natural).[4]

**Figure:**
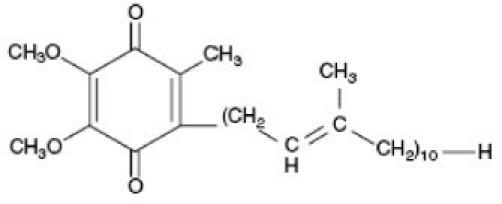
Structure of coenzyme Q_10_.

It is found in every plant and animal cell, and is located in the inner membrane system of the mitochondria, other membranes, and in plasma lipoproteins. The well-recognized function of coenzyme Q_10_ is mitochondrial energy coupling. It is an essential part of the cellular machinery used to produce ATP which provides the energy for muscle contraction and other vital cellular functions. The major part of ATP production occurs in the inner membrane of mitochondria, where coenzyme Q is found. The other important function is that it acts as a primary scavenger of free radicals (FRs) as it is well located in the membranes in close proximity to the unsaturated lipid chains. Less well-established functions include oxidation/reduction control of signal origin and transmission in cells which induce genre expression, control of membrane channels, structure, and lipid solubility.[5]

Bacteria possess several structurally different quinones, among which ubiquinone (UQ), menaquinone (MK) and demethylmenaquinone (DMK) are the most common. These quinones are found in the cytoplasmic membrane, where they participate as electron carriers in respiration and in the disulfide-bond formation. UQ participates in aerobic respiration, whereas MK and DMK have roles in anaerobic respiration. UQ molecules are classified based on the length (*n*) of their isoprenoid side chain (UQ-n). For example, the main UQ species in humans is UQ-10, in rodents it is UQ-9, in *Escherichia coli* it is UQ-8 and, in *Saccharomyces cerevisiae*, it is UQ-6 in varying amounts.[3] Coenzyme Q_9_ is the predominant form in relatively short-lived species such as rats and mice whereas in humans and other long-lived mammals the major homolog is coenzyme Q_10_. Among blood cells, lymphocytes and platelets contain significant amounts of CoQ_10_ whereas red blood cells which lack mitochondria contain only a tiny amount that is likely to be associated with membranes. Lymphocyte CoQ_10_ content can be increased by CoQ_10_ supplementation with concomitant functional improvement as evidenced by enhanced reversal of oxidative DNA damage.[6] The total body pool of CoQ_10_ is estimated to be approximately 0.5–1.5 g in a normal adult.[4] Human cells synthesize CoQ_10_ from the amino acid tyrosine, in an eight-step aromatic pathway, requiring adequate levels of vitamins such as folic acid, niacin, riboflavin, and pyridoxine.[7]

The functions, indications and contraindications of coenzyme Q_10_ are summarized in Table 1.

**Table 1:** A summary of currently recognized functions, indications and contraindications of coenzyme Q_10_

*Functions[1]*	*Indications[2]*	*Contraindications[4]*
Needed for energy conversion (ATP production)	Immune function Periodontal disease	No data have evaluated the safety or toxicity of CoQ_10_ during pregnancy, lactation or childhood. Therefore, its use is not recommended in these patient populations.
	Gastric ulcers	
	Obesity	
An essential antioxidant	Physical performance	
	Muscular dystrophy	All natural products carry the potential for allergic reactions, however, to date, none have been reported with the use of CoQ10.[4]
Regenerates other antioxidants	Allergy	
	Cardiovascular disease: Specific cardiac problems which may benefit include: Cardiomyopathy, congestive heart failure, angina, arrhythmias, prevention of adriamycin toxicity, protection during cardiac surgery, mitral valve prolapse, hypertension, male infertility, diabetes mellitus.	
Stimulates cell growth and inhibits cell death		
Decreased biosynthesis may cause deficiency		

### Coenzyme Q_10_ Deficiency

Since CoQ_10_ is synthesized de novo in all tissues, it is presumed that under normal circumstances they are not dependent on an exogenous supply of CoQ_10_. Although CoQ_10_ can be synthesized “*in vivo*,” situations may arise in which the body’s synthetic capacity is insufficient to meet CoQ_10_ requirements. Susceptibility to CoQ_10_ deficiency appears to be greatest in cells that are metabolically active (such as those in the heart, immune system, gingiva, and gastric mucosa), since these cells presumably have the highest requirements for CoQ_10_.[2]

### A deficiency may result from:[2]

1. Impaired synthesis due to nutritional deficiencies
2. Genetic or acquired defect in synthesis or utilization
3. Increased tissue needs resulting from illness
4. CoQ_10_ levels decline with advancing age

### Assessment of CoQ_10_ status

Plasma or serum CoQ_10_ concentrations are usually employed for the assessment of CoQ_10_ status in humans primarily because of the ease of sample collection. There are several excellent methods based on high pressure liquid chromatography (HPLC) for the analysis of CoQ_10_ in plasma or serum and fasting samples are preferred. Plasma CoQ_10_ concentrations may not necessarily reflect tissue status; it still serves as a useful measure of overall CoQ_10_ status and also as a guide to CoQ_10_ dosing. This is particularly true of degenerative neurologic and muscular diseases where clinical monitoring of plasma CoQ_10_ concentration and also its redox status are highly desirable that could provide valuable information as to the course of treatment. Lymphocyte and platelet CoQ_10_ concentrations could also be considered as potential surrogates for tissue CoQ_10_ status.[6]

### Pharmacokinetics

CoQ_10_ is now recommended as a supplement to traditional therapy for cardiovascular diseases.[8] Though CoQ_10_ is a lipophilic compound, its solubility is extremely limited and the preparations are often characterized by low bioavailability.[9] Absorption of the substance largely depends on its physiochemical characters in the preparation and hence coenzyme Q_10_ in powder, suspension, oil solution, or solubilized form exhibits different bioavailability. Study has shown that solubilized coenzyme Q_10_ is obviously preferred due to its better absorption, higher plasma concentration, and consequently better bioavailability[10] indicating that plasma concentrations of coenzyme Q_10_ are 2–2.5 times higher during long-term oral therapy with solubilized forms[11] and the bioavailability is 3–6 times higher in comparison with powder.[12,13] The pharmacokinetic advantages of solubilized form are responsible for its high efficiency as a cardioprotector: chronic oral treatment led to an increase of not only plasma levels of coenzyme Q_10_ (2.5 times), but of also its concentration in rat myocardium, which improved survival of cardiomyocytes under conditions of ischemia and eventually limited the size of the postinfarction necrotic zone.[10]

## Dosage

The optimal dose of coenzyme Q_10_ is not known, but it may vary with the severity of the condition being treated.[2] Coenzyme Q_10_ is available as a dietary supplement in strengths generally ranging from 15 to 100 mg. In cardiovascular disease patients, CoQ_10_ dosages generally range from 100 to 200 mg per day. Dosages of up to 15 mg/kg/day are being employed in the case of mitochondrial cytopathy patients. A dosage of 600 mg per day was used in the Huntington’s disease trial whereas a dosage of up to 1200 mg per day was employed in the Parkinson’s disease trial.[4]

### Safety of CoQ_10_

CoQ_10_ has an excellent safety record. The safety of high doses of orally ingested CoQ_10_ over long periods is well documented in human subjects and also by chronic toxicity studies in animals. The side effects reported in human studies are generally limited to mild gastrointestinal symptoms such as nausea and stomach upset seen in a small number of subjects. No adverse effects were observed with daily doses ranging from 600 to 1200 mg in two trials on Huntington’s and Parkinson’s diseases. More recent data document the safety and tolerability of CoQ_10_ at doses as high as 3000 mg per day in patients with Parkinson’s disease and amyotrophic lateral sclerosis.[4]

### CoQ_10_ and periodontitis

Chronic periodontitis is the direct result of accumulation of subgingival plaque. The microflora of this plaque is extremely complex causing problems in establishing which organisms are responsible for tissue destruction associated with the disease. Despite these problems, there is one point on which investigators agree, the subgingival flora of healthy gingival crevice is sparse and consists largely of aerobic and facultative bacteria, while in diseased state there is an increase in the proportion of anaerobic bacteria. These bacteria cause the observed tissue destruction directly by toxic products and indirectly by activating host defense systems, i.e. inflammation.[14] Inflammation represents the response of the organism to a noxious stimulus, whether mechanical, chemical, or infectious. It is a localized protective response elicited by injury or destruction of tissues, which serves to destroy, dilute, or wall off both the injurious agent and the injured tissue. Whether acute or chronic, inflammation is dependent upon regulated humoral and cellular responses, and the molecules considered to mediate inflammation at one time or another are legion.[1] However, an event characteristic of mammalian inflammation, tissue infiltration by polymorphonuclear leukocytes and monocytes and subsequent phagocytosis features non-mitochondrial O_2_ consumption, which may be 10 or 20 times that of resting consumption ultimately ends in generating free radicals (FRs) and reactive oxygen species (ROS), such as superoxide anion radicals, hydrogen peroxide, hydroxyl radicals, and hypochlorous acid, all capable of damaging either cell membranes or associated biomolecules.[14] Because of their high reactivity, several FRs and ROS can rapidly modify either small, free biomolecules (i.e., vitamins, amino acids, carbohydrates, and lipids) or macromolecules (i.e., proteins, nucleic acids) or even supramolecular structure (i.e., cell membranes, circulating lipoproteins). The type and the extent of damage depend upon the site of generation. Usually, the oxidative damage is perfectly controlled by the anti-oxidant defense mechanisms of the surrounding tissues but plaque microorganisms promoting periodontitis can unbalance this equilibrium. A massive neutrophil migration to the gingiva and gingival fluid leads to abnormal spreading of FR/ROS produced. Consequently, this led to a search for appropriate “antioxidant therapy” in inflammatory periodontal disease.[14]

A deficiency of coenzyme Q_10_ at its enzyme sites in gingival tissue may exist independently of and/or because of periodontal disease. If a deficiency of coenzyme Q_10_ existed in gingival tissue for nutritional causes and independently of periodontal disease, then the advent of periodontal disease could enhance the gingival deficiency of coenzyme Q_10_.[15] In such patients, oral dental treatment and oral hygiene could correct the plaque and calculus, but not that part of the deficiency of CoQ_10_ due to systemic cause; therapy with CoQ_10_ can be included with the oral hygiene for an improved treatment of this type of periodontal disease.[15]

The specific activity of succinic dehydrogenase–coenzyme Q_10_ reductase in gingival tissues from patients with periodontal disease against normal periodontal tissues has been evaluated using biopsies, which showed a deficiency of CoQ_10_ in patients with periodontal disease. On exogenous CoQ_10_ administration, an increase in the specific activity of this mitochondrial enzyme was found in deficient patients.[15–18] The periodontal score was also decreased concluding that CoQ_10_ should be considered as an adjunct for the treatment of periodontitis in current dental practice.[19]

Not only succinate dehydrogenase CoQ_10_ reductase, but also succinate cytochrome c reductase and NADH cytochrome c reductase showed decreased specific activity in periodontitis patients.[20] On exogenous administration of CoQ_10_ showed improved specific activity of these enzymes with significant reduction of motile rods and spirochetes.[21] The preliminary data indicated that CoQ_10_ may reduce gingival inflammation without affecting GCF total antioxidant levels,[22] whereas one more study showed significant reduction in TBRAS in GCF in patients treated with scaling and root planning with CoQ_10_.[23]

Topical application of CoQ_10_ to the periodontal pocket was evaluated with and without subgingival mechanical debridement. In the first three-week period, significant reduction in gingival crevicular fluid flow, probing depth and attachment loss were found and significant improvements in modified gingival index, bleeding on probing and peptidase activity derived from periodontopathic bacteria were observed only at experimental sites (CoQ_10_ with subgingival mechanical debridement).[24] It suggested that the research literature on coenzyme Q_10_ ’s periodontal effect does not extend to International English language dental literature. The review of available literature does not give any ground for the claims regarding benefit of coenzyme Q_10_ and has no place in periodontal treatment.[25]

A study evaluated the periodontium condition after oral applications of coenzyme Q_10_ with vitamin E. The total antioxidant status (TAS) in the mixed saliva by the colorimetric method was determined twice. The average value of plaque index decreased from 1.0 to 0.36, average value of interdental hygiene index was reduced from 39.51–6.97%, gingival index values decreased from 0.68 to 0.18, and the values of sulcus bleeding index decreased from 7.26 to 0.87. Periodontal pockets also shallowed by 30%. The laboratory examination result improved by 20%. It concluded that coenzyme Q_10_ with vitamin E had a beneficial effect on the periodontal tissue.[26]

Because it is an antioxidant, coenzyme Q_10_ has received much research attention in the medical literature in the last several years. Although coenzyme Q_10_ may have been viewed as an alternative medication, it is used routinely, both topically and systemically, by many believing dentists and periodontists. However, there is a dearth of new information for coenzyme Q_10_ in the treatment of periodontal conditions. A deficiency of CoQ_10_ has been found in the gingiva of patients with periodontal disease.[15,16] Gingival biopsies from patients with inflamed periodontal tissues showed a deficiency of CoQ_10_, in contrast to patients with normal periodontal tissues. Many clinical trials with oral administration of CoQ_10_ to patients with periodontal disease have been conducted. The results have shown that oral administration of CoQ_10_ increases the concentration of CoQ10 in the diseased gingiva and effectively suppresses advanced periodontal inflammation[17,27,28] and periodontal microorganisms. Clinical study with interpocket application has shown CoQ_10_ is an effective adjunctive in the treatment of chronic periodontitis and also found to enhance the resistance of the periodontal tissues to periodontopathic bacteria (unpublished data).

## Conclusion

The concept of ROS-induced destruction has led to search for an appropriate complimentary antioxidant therapy in the treatment of numerous diseases including inflammatory periodontal diseases. Because it is an antioxidant, there is a dearth of new information for coenzyme Q_10_ in the treatment of periodontal conditions. The pharmacology of coenzyme Q_10_ indicates that it may be an agent for treatment of periodontitis. On the basis of on new concepts of synergism with nutritional supplements and host response, coenzyme Q_10_ may possibly be effective as a topical and/or systemic role or adjunctive treatment for periodontitis either as a stand-alone biological or in combination with other synergistic antioxidants (i.e., vitamins C and E).
